# Biomarkers of Hypercoagulability and Thromboinflammation in Cervical Cancer-Associated Thrombosis: A Systematic Review with Translational Insights from Breast Cancer

**DOI:** 10.3390/ijms27167302

**Published:** 2026-08-16

**Authors:** Dana Taizhanova, Nurgul Serikbay, Pedro Henrique Fernandes do Carmo Las Casas, Jovana Mijucic, Bodaubay Roza, Dmitry Zubkov, Diana Toleubekova, Veronica Bovt, Zoubida Tazi Mezalek, Patrick Vandreden, Mohammed A. Baghdadi, Nida Saleem, Alfonso Tafur, Eleftheria Elmina Lefkou, Fakiha Siddiqui, Prakasha Kempaiah, Jawed Fareed, Victoria Bitsadze, Grigoris T. Gerotziafas

**Affiliations:** 1 Department of Internal Diseases, NJSC Karaganda Medical University, 100000 Karaganda, Kazakhstan; taizhanova_kgma@mail.ru (D.T.); bovt.veronika.1995@gmail.com (V.B.); 2 Multidisciplinary Hospital No. 1, 100000 Karaganda, Kazakhstan; toleubekovadiana@gmail.com; 3 Clinic of Karaganda Medical University, 100000 Karaganda, Kazakhstan; 4 CaVITE Research Team, Consultation Thrombosis in Oncology (CoThON), Saint-Antoine Research Centre (CRSA), INSERM UMR_S_938, Saint-Antoine University Hospital, University Institute of Cancerology (UIC), Assistance Publique Hôpitaux de Paris (AP-HP), Sorbonne University, 75012 Paris, France; pedrohlascasas@gmail.com (P.H.F.d.C.L.C.); jovana.mijucic@gmail.com (J.M.); nida.saleem@lumc.edu (N.S.); elefkou@uth.gr (E.E.L.); grigorios.gerotziafas@inserm.fr (G.T.G.); 5 Clinic for Medical Oncology, Institute for Oncology and Radiology of Serbia, 11000 Belgrade, Serbia; 6 Department of Obstetrics, Gynecology and Perinatology, NJSC Karaganda Medical University, 100000 Karaganda, Kazakhstan; zubkov@qmu.kz; 7 Department of Clinical Haematology, Mohammed V University, Rabat 10000, Morocco; z.tazimezalek@gmail.com; 8 Diagnostica Stago, 92230 Gennevilliers, France; 9 Thrombosis and Hemostasis Research Laboratories, Centre of Translational Research and Education, Department of Pathology and Laboratory Medicine, Loyola University Stritch School of Medicine, Maywood, IL 60153, USA; fsiddiqui3@luc.edu (F.S.); prakashamk@gmail.com (P.K.); jfareed@luc.edu (J.F.); 10 Department of Medicine, Vascular Medicine, Endeavor Health, Evanston, Pritzker School of Medicine, University of Chicago, Chicago, IL 60637, USA; fonz.tafur@gmail.com; 11 Blood Transfusion and Haemostasis Unit, Faculty of Medicine, School of Health Sciences, University of Thessaly, 41334 Larissa, Greece; 12 Thrombosis Centre, Department of Obstetrics, Gynecology and Perinatal Medicine, I.M. Sechenov First Moscow State Medical University (Sechenov University), 119991 Moscow, Russia; vikabits@mail.ru; 13 VAS—European Independent Foundation in Angiology/Vascular Medicine, 20122 Milan, Italy

**Keywords:** cancer-associated thrombosis, cervical cancer, breast cancer, biomarkers, hypercoagulability, thromboinflammation, precision medicine, extracellular vesicles, coagulome, venous thromboembolism

## Abstract

Cancer-associated thrombosis (CAT) is a major cause of morbidity and mortality in patients with malignancy. Biomarkers of hypercoagulability and thromboinflammation may improve risk stratification and support personalised thromboprophylaxis, but evidence remains heterogeneous, particularly in cervical cancer. A systematic review was conducted according to PRISMA 2020. PubMed/MEDLINE, Scopus, Embase, and Web of Science were searched for studies published between January 2009 and March 2025 evaluating biological, molecular, genetic, and imaging biomarkers associated with hypercoagulability and thromboinflammation in women withcervical cancer. Evidence from breast cancer and broader CAT studies was incorporated to provide translational context. Owing to substantial methodological heterogeneity, findings were synthesised qualitatively. Twenty-five cervical cancer studies met eligibility criteria. D-dimer was the most extensively investigated biomarker and was consistently associated with VTE risk, although specificity was limited. Biomarkers of thrombin generation and fibrinolytic activation, including thrombin–antithrombin complexes, prothrombin fragment 1+2, and plasmin–α2-antiplasmin complex, demonstrated greater mechanistic specificity. Multimarker panels integrating coagulation, fibrinolysis, endothelial injury, platelet activation, and inflammation showed superior predictive performance compared with single biomarkers. Emerging biomarkers, including circulating tumour DNA, extracellular vesicles, and microRNAs, further supported tumour-driven thromboinflammation. SERPINE1 and F2 gene variants were associated with thrombotic risk and adverse prognosis. Current evidence supports further evaluation of integrated multimodal biomarker strategies for CAT risk assessment, but prospective, standardised, tumour-specific studies are required before biomarker-guided approaches can be implemented in clinical practice.

## 1. Introduction

### 1.1. Clinical Context and Epidemiological Burden

Cancer-associated thrombosis (CAT) is the second leading cause of death in patients with active malignancy, exceeded only by cancer progression itself, and represents a major source of treatment-related morbidit [1,2,3,4,5] Venous thromboembolism (VTE)—encompassing deep vein thrombosis (DVT) and pulmonary embolism (PE)—occurs approximately 3- to 9-fold more frequently in cancer patients than in the general population, with the highest absolute risk concentrated in the first year following diagnosis or disease recurrence [1,6]. Reported VTE incidence varies widely, reaching up to 20% depending on tumour type, disease stage, anticancer treatment modality, and patient-related risk factors [5].

Beyond its direct clinical consequences, CAT substantially complicates oncological management. Thrombotic events frequently necessitate delays, dose reductions, or discontinuation of systemic anticancer therapy and complicate perioperative decision-making [3,4,5]. Long-term anticoagulant therapy, while essential for secondary CAT prevention, increases the risk of major or clinically relevant non-major bleeding—a particular concern in patients with thrombocytopenia, mucosal tumours, renal insufficiency, or treatment-related toxicities [3,4,5]. The progressive shift toward ambulatory cancer care has further amplified the proportion of VTE events occurring outside hospital settings, reinforcing the clinical imperative for accurate, dynamic, and individualised thrombosis risk stratification [1]. CAT also imposes a substantial economic burden through increased hospitalisation rates, prolonged treatment courses, and escalating resource utilisation [7].

### 1.2. Pathophysiology: A Dynamic Thromboinflammatory Process

The pathophysiology of CAT involves a complex and temporally dynamic interplay between tumour-intrinsic procoagulant activity, systemic inflammation, endothelial cell injury, platelet activation, and dysregulation of fibrinolytic pathways [1,6,8,9]. Cancer cells promote coagulation activation principally through tissue factor (TF) overexpression, release of TF-bearing procoagulant extracellular vesicles (EVs), direct platelet and endothelial cell engagement, and oncogenic modulation of fibrinolytic regulators [8,9]. In parallel, pro-inflammatory cytokines—notably interleukin-6 (IL-6)—and innate immune effectors, including neutrophil extracellular traps (NETs), amplify thrombin generation and vascular injury through immunothrombotic mechanisms, linking tumour progression to systemic haemostatic dysregulation [8,9].

An emerging dimension in CAT pathophysiology is the concept of the tumour ‘coagulome’: cancer cell-intrinsic transcriptional programmes that collectively regulate expression of coagulation factors, fibrinolytic modulators, and procoagulant extracellular components [8,9]. Circulating molecular entities—including ctDNA [10], circulating tumour cells (CTCs) and platelet-tumor interactions [11] EVs [12,13,14,15] and microRNAs [16] may contribute to systemic thromboinflammatory responses and reflect tumour burden, metastatic potential, and biological aggressiveness. Importantly, tumour-associated hypercoagulability exhibits substantial heterogeneity across cancer types and disease stages, supporting a model in which distinct cancer-specific thromboinflammatory phenotypes emerge from the interplay of oncogenic signalling, host inflammatory response, and treatment-related vascular toxicity [8,9].

### 1.3. The Challenge of Balancing Thrombosis and Bleeding Risk

Despite anticoagulant therapy, patients with CAT remain at substantial risk of recurrent VTE, with recurrence rates estimated at 5–10% within six months and exceeding 10% at 12 months in patients with advanced disease or ongoing anticancer treatment [16,17,18]. This persistent thrombotic risk reflects sustained tumour-driven hypercoagulability, residual endothelial activation, and dysregulated thromboinflammatory signalling [8,9].

Conversely, anticoagulant therapy is associated with major bleeding rates of approximately 3–6% within the first six months in CAT populations, particularly in patients with specific tumour types, thrombocytopenia, or organ dysfunction [16,17]. These competing risks highlight the fundamental limitation of static clinical risk models and underscore the need for dynamic, biomarker-based approaches capable of identifying patients with persistent hypercoagulability, active thromboinflammation, or elevated bleeding susceptibility in real time [9].

### 1.4. Sex-Specific Considerations and Underrepresentation of Female Malignancies

Optimising CAT prevention and management in women carries particular clinical importance given the global burden of female malignancies and the potential influence of sex-specific biological and treatment-related factors on thrombotic risk [1,3,4,5].Breast and cervical cancers differ in treatment exposure and tumour-associated haemostatic mechanisms, which may contribute to differences in thrombotic risk and biomarker expression [9]. Cervical cancer disproportionately affects women in low- and middle-income settings, where delayed diagnosis, advanced disease at presentation, and limited access to thrombosis prevention strategies may further contribute to CAT-related morbidity.

Despite these considerations, women—and particularly those with gynaecological malignancies—remain underrepresented in biomarker-driven CAT studies. Sex-specific mechanisms of cancer-associated hypercoagulability are insufficiently characterised, and validated precision thrombosis strategies adapted to female cancers are largely absent from current clinical frameworks [9].

### 1.5. Limitations of Current Thrombosis Risk Assessment

Existing risk scoring systems for CAT, including the Khorana score [19] and its extensions (e.g., the Vienna Cancer and Thrombosis Study [CATS] score [20], were developed primarily in heterogeneous mixed-cancer populations and demonstrate only moderate discriminatory performance in individual tumour types. These models incorporate few haemostatic or inflammatory biomarkers and do not capture the dynamic thromboinflammatory processes that evolve throughout disease progression and treatment [21]. Emerging evidence consistently demonstrates that single-biomarker approaches are insufficient to reflect the biological complexity of CAT [22,23,24,25,26].

Multimarker strategies integrating coagulation activation, fibrinolysis, endothelial dysfunction, inflammatory signalling, and tumour-derived molecular signatures have shown promising improvements in risk stratification compared with isolated biomarkers, supporting a shift toward biologically informed, multidimensional assessment frameworks [9].

### 1.6. Rationale and Objectives of the Present Review

Despite growing evidence supporting the role of biomarkers in CAT risk stratification, the available data remain fragmented, methodologically heterogeneous, and largely unvalidated in underrepresented malignancies such as cervical cancer. Breast cancer has been more extensively investigated and provides an important translational reference for understanding tumour-associated hypercoagulability, thromboinflammatory signalling, and biomarker performance [9].

These two malignancies share relevant mechanisms, including inflammatory tumour microenvironments, endothelial activation, platelet–tumour interactions, and coagulome-associated pathways, while differing substantially in tumour biology, treatment exposure, and thrombotic phenotype, providing a unique comparative framework. Importantly, this comparison does not imply that cervical and breast cancers share identical oncogenic drivers or molecular architectures. Cervical cancer is largely shaped by HPV-driven viral oncoproteins, which influence immune evasion, chronic inflammation, angiogenesis, endothelial activation, and tissue remodelling, whereas breast cancer-associated thrombosis may be influenced by hereditary susceptibility, hormone-related signalling, molecular subtype, tumour proliferation, metastatic burden, and treatment exposure. Nevertheless, these distinct upstream mechanisms may converge on common vascular and thromboinflammatory pathways relevant to CAT, including chronic inflammatory signalling, endothelial injury, platelet–tumour interactions, tissue factor expression, extracellular vesicle release, fibrinolytic imbalance, and coagulation activation. Accordingly, breast cancer was used in this review not as a biologically equivalent comparator to cervical cancer, but as a translational reference model to contextualise shared vascular mechanisms and to distinguish potentially common thromboinflammatory biomarker signals from tumour-specific molecular drivers.

This systematic review was therefore designed as a critical evaluation of biological, molecular, and genetic biomarkers associated with hypercoagulability and thromboinflammation in women with cervical cancer, complemented by translational comparative evidence from breast cancer to contextualise mechanistic and biomarker-related findings.

Specifically, this review aims to assess the role of biomarkers in: (i) CAT risk stratification; (ii) CAT diagnosis; (iii) prediction of recurrent VTE; and (iv) identification of elevated bleeding risk. By integrating evidence across haemostatic, inflammatory, endothelial, molecular, and genetic domains, this review aims to support the development of standardised, multimodal, and biologically informed approaches for biomarker-driven precision management of CAT in women.

## 2. Methods

### 2.1. Study Design and Reporting Framework

This study was conducted as a systematic review with translational narrative integration, in accordance with the Preferred Reporting Items for Systematic Reviews and Meta-Analyses (PRISMA) 2020 statement [22]. The research question was structured using a modified PICO framework adapted for biomarker-based translational research:

Population (P): Adult women with cervical cancer, irrespective of disease stage or treatment setting (curative, adjuvant, neoadjuvant, perioperative, ambulatory, or palliative). Studies involving paediatric populations, non-malignant conditions, or male-only cohorts were excluded.

Index (I): Biological, molecular, genetic, haemostatic, inflammatory, and endothelial biomarkers associated with hypercoagulability and thromboinflammation in the context of CAT risk stratification, diagnosis, recurrence prediction, or bleeding-risk assessment.

Comparator (C): Where available, biomarker profiles were compared between patients with and without thrombotic events or across predefined thromboticrisk categories. Studies lacking comparator groups were not excluded given the exploratory nature of several biomarker investigations.

Outcomes (O): Objectively confirmed VTE (DVT and/or PE), recurrent VTE, VTE-related mortality, and other clinically relevant thrombotic complications. Arterial thrombotic events (ATEs) were included when explicitly reported.

For the primary systematic component, CAT was defined as VTE occurring in patients with active cervical cancer [27]. Breast cancer studies were incorporated exclusively as secondary translational comparators to support comparative mechanistic interpretation. The review protocol was not prospectively registered in PROSPERO; however, the eligibility criteria and analytical framework were established a priori before study selection.

### 2.2. Search Strategy

A comprehensive literature search was performed in PubMed/MEDLINE, Scopus, Embase, and Web of Science for studies published between January 2009 and March 2025 (final search: March 2025). The strategy combined controlled vocabulary terms (MeSH, Emtree) with free-text keywords relating to cervical cancer, breast cancer, CAT, VTE, hypercoagulability, thromboinflammation, coagulation biomarkers, EVs, TF, ctDNA, and related molecular biomarkers, connected by Boolean operators. An example PubMed query is provided in Supplementary Table S1. Reference lists of eligible studies and relevant reviews were manually screened for additional records. Only peer-reviewed full-text publications in English or Russian were considered.

### 2.3. Study Selection and Eligibility Criteria

Titles and abstracts were screened against predefined eligibility criteria; potentially relevant records underwent full-text assessment. Discrepancies were resolved by consensus.

Eligible study designs included prospective and retrospective cohort studies, case–control studies, translational biomarker investigations, and genetic association studies evaluating CAT-related biomarkers in women with cervical cancer. Clinical studies were required to report objectively confirmed thrombosis-related outcomes. Mechanistic and translational studies involving breast cancer or broader cancer populations were included exclusively for comparative biological interpretation.

Excluded studies: Narrative reviews, systematic reviews, meta-analyses, editorials, conference abstracts, case reports, animal and in vitro studies, studies without thrombosis-related outcomes, and studies lacking biomarker data or adequate methodological detail (Supplementary Table S2).

**Figure 1:**
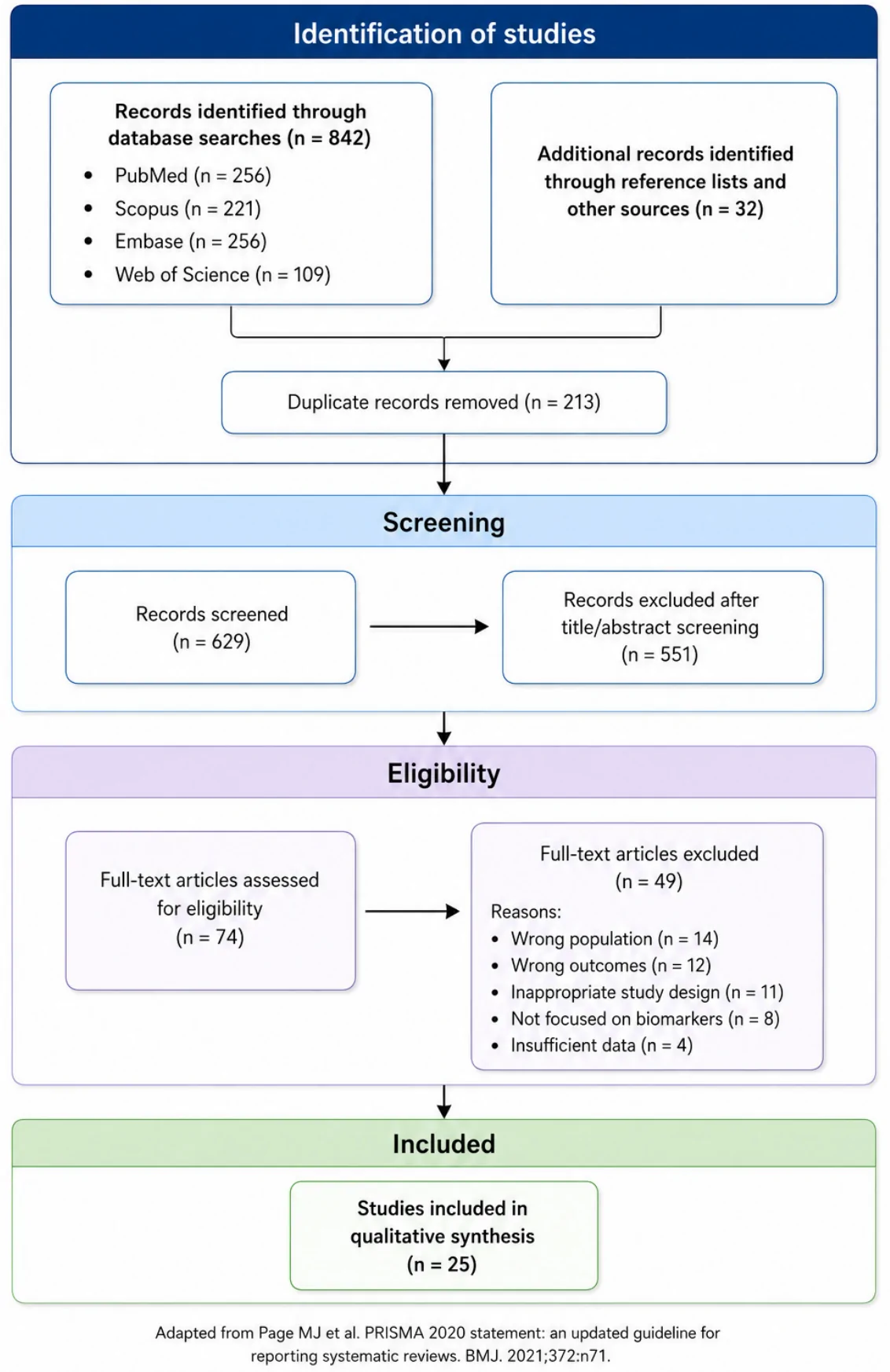
PRISMA 2020 flow diagram of study identification, screening, eligibility assessment, and inclusion process [22].

Database searches identified 842 records; 32 additional records were retrieved through manual screening. After deduplication, 629 records underwent title and abstract screening; 74 full-text articles were assessed for eligibility; 25 studies met the criteria and were included in the qualitative synthesis. The study selection process is depicted in Figure 1 (PRISMA 2020 flow diagram).

### 2.4. Data Extraction

Data extraction was performed independently by two reviewers using a standardised predefined framework. The extracted variables included study characteristics (design, country, year, sample size), patient characteristics (cancer type, disease stage, treatment setting), biomarker domain and definitions, thrombotic outcome definitions and incidence, follow-up duration, and principal clinical and mechanistic findings. Discrepancies were resolved by consensus.

### 2.5. Quality Assessment

The methodological quality of the eligible observational studies was assessed using the Newcastle–Ottawa Scale (NOS), evaluating selection, comparability, and outcome/exposure assessment (maximum score: 9). Studies scoring 7–9 were classified as high quality, 5–6 as moderate quality, and <5 as low quality. NOS was not applied to mechanistic, translational, or review-based references (Supplementary Table S3).

### 2.6. Data Synthesis

Given the substantial heterogeneity in study design, populations, biomarker assays, cut-off values, and outcome definitions, quantitative meta-analysis was not performed. Although D-dimer was the most frequently investigated biomarker and was therefore specifically considered for potential quantitative synthesis, pooling was judged inappropriate because of substantial variation in assay methods, cut-off definitions, timing of biomarker measurement, disease stage, treatment setting, follow-up duration, and VTE outcome definitions. Accordingly, D-dimer findings were synthesised qualitatively, with emphasis on consistency of association, specificity limitations, and clinical interpretability. Overall, findings were synthesised according to biological biomarker domain, thromboinflammatory pathway, clinical application, and comparative translational evidence between cervical and breast cancer (Supplementary Table S4).

## 3. Results

### 3.1. Overview of Included Studies

Twenty-five studies met eligibility criteria and were included in the qualitative synthesis (Table 1). Study designs were predominantly observational, comprising prospective and retrospective cohort studies, case–control investigations, population-based analyses, and genetic association studies. The evidence base spanned a broad spectrum of biomarker domains: conventional coagulation markers (D-dimer, fibrinogen, prothrombin time), specific haemostatic biomarkers (TAT, F1+2, PIC, P-selectin, thrombomodulin), inflammatory mediators (CRP, IL-6), and emerging molecular biomarkers (microRNAs, EVs, coagulome-associated genetic variants).

Substantial heterogeneity was observed across studies in terms of patient population, disease stage, biomarker selection, analytical methodology, and outcome definitions. Stage distribution represented a particularly important source of heterogeneity, as the included studies enrolled patients across localised, locally advanced, operable, metastatic, perioperative, and treatment-exposed settings. Because biomarker expression may vary according to tumour burden, metastatic dissemination, treatment exposure, and inflammatory activity, stage-specific interpretation was limited by the design and reporting of the available studies. This precluded quantitative meta-analysis; findings were therefore synthesised using a qualitative translational framework. The principal study characteristics are summarised in Table 1.

The table summarises observational studies investigating circulating, cellular, molecular, and tumour-related biomarkers associated with venous thromboembolismin patients with cervical cancer. The majority of studies identified biomarkers of hypercoagulability, inflammation, platelet activation, endothelial dysfunction, and tumour aggressiveness as significant predictors of VTE occurrence. D-dimer is the most extensively investigated biomarker, while fibrinogen, platelet count, plasmin–α2 plasmin inhibitor complex (PIC), soluble P-selectin (sP-selectin), tissue factor (TF), vascular endothelial growth factor (VEGF), microRNAs, and inflammatory indices have emerged as promising complementary markers for thrombosis risk stratification in cervical cancer patients.

### 3.2. Biological Domains of Cancer-Associated Hypercoagulability

Biomarkers were categorised according to their predominant pathophysiological role across six domains: (1) coagulation activation and fibrinolysis, (2) inflammation and thromboinflammation, (3) platelet activation, (4) endothelial dysfunction, (5) tumour-derived molecular biomarkers, and (6) genetic determinants of thrombogenesis (Table 2). These domains are highly interconnected and collectively contribute to the tumour-associated thromboinflammatory syndrome driving CAT.

#### 3.2.1. Coagulation and Fibrinolysis Biomarkers

D-dimer was the most extensively investigated biomarker and was consistently associated with increased VTE risk across both breast and cervical cancer cohorts [20,22,23,24,49,50,51]. However, its diagnostic specificity was substantially limited by tumour burden, systemic inflammation, endothelial injury, and treatment-related hypercoagulability, resulting in frequent elevation in the absence of objectively confirmed thrombosis [9,50].

Biomarkers such as TAT complexes and prothrombin fragment 1+2 (F1+2) more directly reflect ongoing thrombin generation and haemostatic activation than D-dimer alone [22,24,25]. The plasmin–α2-antiplasmin complex (PIC), a marker of fibrinolytic activation, was frequently elevated in CAT and demonstrated improved diagnostic utility when integrated with coagulation biomarkers [23,25,26]. Multimarker panels combining D-dimer, TAT, PIC, thrombomodulin (TM), and t-PAICshowed improved diagnostic and predictive performance compared with individual biomarkers, supporting the concept that CAT reflects multidimensional haemostatic dysregulation [24,25,26].

#### 3.2.2. Inflammatory Biomarkers

Elevated CRP, hs-CRP, and IL-6 were associated with increased VTE risk, particularly in patients with advanced-stage cancer and persistent systemic inflammatory activation [52,53]. Concomitant elevation of inflammatory and coagulation biomarkers across multiple studies supported the central role of coordinated inflammatory–haemostatic dysregulation in CAT pathogenesis, consistent with tumour-driven cytokine release, TF upregulation, and amplification of thrombin generation through immunothrombotic pathways [9].

#### 3.2.3. Platelet Activation and Endothelial Dysfunction

Elevated soluble P-selectin, reflecting enhanced platelet activation and platelet–endothelial interactions, was associated with increased risk of incident and recurrent VTE [9,21,53]. Endothelial dysfunction markers—TM and t-PAIC—were frequently elevated in patients with VTE across multiple cancer populations [9,24,25]. suggesting that endothelial injury acts synergistically with inflammatory and coagulation pathways to promote vascular injury, thrombin generation, and CAT. Collectively, these findings support a model in which vascular homeostasis disruption—encompassing coordinated endothelial, platelet, inflammatory, and haemostatic activation—underlies CAT development [9]. 

#### 3.2.4. Molecular and Tumour-Derived Biomarkers

Circulating tumour DNA (ctDNA) was associated with increased VTE risk in pan-cancer cohorts and appeared to reflect tumour burden, biological aggressiveness, and systemic prothrombotic activity [10]. MicroRNAs were implicated in the post-transcriptional modulation of endothelial activation, platelet signalling, inflammatory pathways, and coagulation-related gene expression, although their clinical utility remains exploratory [16,26].

TF-positive EVs and cancer cell-derived EVs, bearing procoagulant TF or phosphatidylserine, were associated with enhanced thrombin generation and VTE occurrence [12,13,14,15,17]. These vesicles contribute to systemic hypercoagulability through endothelial activation, platelet engagement, and amplification of coagulation signalling [12,14,15,17]. CTCs were also implicated in thrombogenesis through direct interactions with platelets and endothelial cells [11]. Tumour-specific coagulome signatures—including upregulation of TF and SERPINE1-related pathways—further supported the mechanistic link between oncogenic signalling and coagulation activation [9,12,24,25].

### 3.3. Biomarkers for CAT Risk Stratification

#### 3.3.1. Breast Cancer

Elevated D-dimer, fibrinogen, factor VIII, and altered thrombin generation parameters were associated with increased VTE risk in breast cancer populations, though effect sizes and predictive performance varied across studies [47,48,54]. Integrated multimarker models demonstrated superior predictive performance: in a prospective multicentre cohort study of metastatic breast cancer, a model combining Ki-67 and fibrinogen stratified patients into risk tiers, with a VTE incidence of approximately 2% in the low-risk group versus 13% in the high-risk group [54], underscoring the additive value of tumour proliferative activity and haemostatic activation. Emerging molecular biomarkers—including EVs, CTCs, ctDNA, and microRNAs—provided additional mechanistic insights, although none have been prospectively validated for routine clinical implementation [10,11,12,13,14,15,16,17].

#### 3.3.2. Cervical Cancer

Evidence supporting CAT risk stratification in cervical cancer derives predominantly from observational cohorts, perioperative studies, chemoradiotherapy-associated investigations, and genetic association analyses [18,19,21,23,24,25]. D-dimer was most frequently evaluated and systematically elevated in VTE cases, but specificity remained limited due to tumour burden, disease stage, and treatment effects [18,21].

Biomarkers reflecting in vivo thrombin generation and fibrinolytic dysregulation—notably TAT complexes and PIC—provided greater mechanistic insight and improved thromboticrisk discrimination [19,21,23]. Multimarker panels integrating coagulation, fibrinolysis, and endothelial dysfunction markers demonstrated improved thromboticrisk discrimination compared with individual biomarkers [21,23]. Genetic variants in SERPINE1 and F2 were associated with thrombotic risk and adverse prognosis [24,25], supporting the concept that tumour-specific molecular and genetic signatures contribute directly to cancer-induced hypercoagulability. Emerging molecular biomarkers (ctDNA, microRNAs, EVs) remain insufficiently validated in cervical cancer but represent promising precision-medicine candidates [10,12,13,14,15,16,17,26].

### 3.4. Clinical Utility of Biomarkers for Diagnosis and Recurrence Monitoring

#### 3.4.1. Breast Cancer

In breast cancer, no single biomarker has demonstrated sufficient accuracy for the diagnosis of CAT. Although D-dimer remains highly sensitive, its specificity is limited because elevated levels frequently reflect tumour burden, systemic inflammation, or ongoing anticancer treatment rather than thrombosis alone [47,53,54,55]. Consequently, multimarker approaches integrating coagulation and inflammatory biomarkers have been proposed to improve diagnostic performance, although none has yet been prospectively validated for routine clinical practice [48,51,52,54].

Patients with active breast cancer remain at substantial risk of recurrent VTE despite anticoagulant therapy, reflecting persistent thromboinflammatory activation [53]. The HISPALIS study demonstrated that persistent elevation of D-dimer, hs-CRP, and P-selectin after discontinuation of low-molecular-weight heparin was associated with recurrent thrombotic events, suggesting that residual biomarker activation may identify patients requiring closer clinical surveillance [52]. However, no biomarker-guided strategy has yet been validated to individualise anticoagulation duration in breast cancer-associated thrombosis [53,55].

#### 3.4.2. Cervical Cancer

In cervical cancer, evidence supporting the clinical application of biomarkers for CAT diagnosis remains limited. Current studies indicate that combining biomarkers reflecting thrombin generation, fibrinolysis, and endothelial dysfunction—particularly TAT, PIC, TM, and t-PAIC—provides greater diagnostic value than individual biomarkers alone [19,21,23]. Among these, PIC appears particularly promising for perioperative risk assessment in gynaecological oncology, although validation in larger prospective cohorts is still required [23].

Evidence regarding recurrence monitoring in cervical cancer is scarce and is largely extrapolated from broader oncology populations [53,55]. Although genetic determinants such as SERPINE1 and F2 variants have been associated with thrombotic susceptibility and clinical outcomes [24,25], their role in predicting recurrent CAT or guiding anticoagulant management has not yet been established. Prospective tumour-specific studies are therefore needed to define the clinical utility of biomarker-guided diagnosis, recurrence monitoring, and treatment individualisation.

### 3.5. Comparative Evidence: Breast Versus Cervical Cancer

Comparative analysis revealed shared thromboinflammatory mechanisms and important tumour-specific differences influencing CAT biology, biomarker performance, and clinical applicability.

Across both malignancies, CAT-related pathways included coagulation activation, endothelial dysfunction, platelet activation, inflammatory signalling, and tumour-derived procoagulant activity mediated through tissue factor expression and extracellular vesicles [8,9,12,13,14,15,17,18,19,20,21,23,54]. These findings reinforce the concept that CAT represents a multidimensional interaction between tumour biology and the host haemostatic and inflammatory response.

The maturity of biomarker research and translational validation differed substantially between the two malignancies. In breast cancer, haemostatic and tumour-related biomarkers have been investigated within more advanced risk-assessment frameworks. In particular, a prospective study demonstrated the potential value of combining fibrinogen with the proliferative tumour marker Ki-67 for VTE risk stratification [54]. Additional studies have evaluated D-dimer, recurrence-related biomarkers, and persistent thromboinflammatory activation in breast cancer and broader cancer-associated thrombosis populations [47,51,52,53].

Molecular biomarker research—including ctDNA, extracellular vesicles, tissue factor-bearing microparticles, and microRNAs—has also been more extensively developed in breast cancer and broader translational oncology settings [10,11,12,13,14,15,16,17]. In contrast, evidence in cervical cancer remains comparatively limited, fragmented, and predominantly observational, with most studies focusing on coagulation activation, fibrinolysis, endothelial dysfunction, inflammation, and genetic susceptibility [18,19,20,21,23,24,25,26].

Accordingly, conclusions regarding biomarker performance in cervical cancer were derived primarily from cervical cancer-specific studies, whereas evidence from breast cancer and broader oncology populations was used to provide translational context rather than direct clinical validation. This distinction is important because biomarker expression, thrombotic risk, and clinical utility may vary according to tumour type, disease stage, treatment exposure, tumour burden, and underlying molecular drivers.

**Figure 2:**
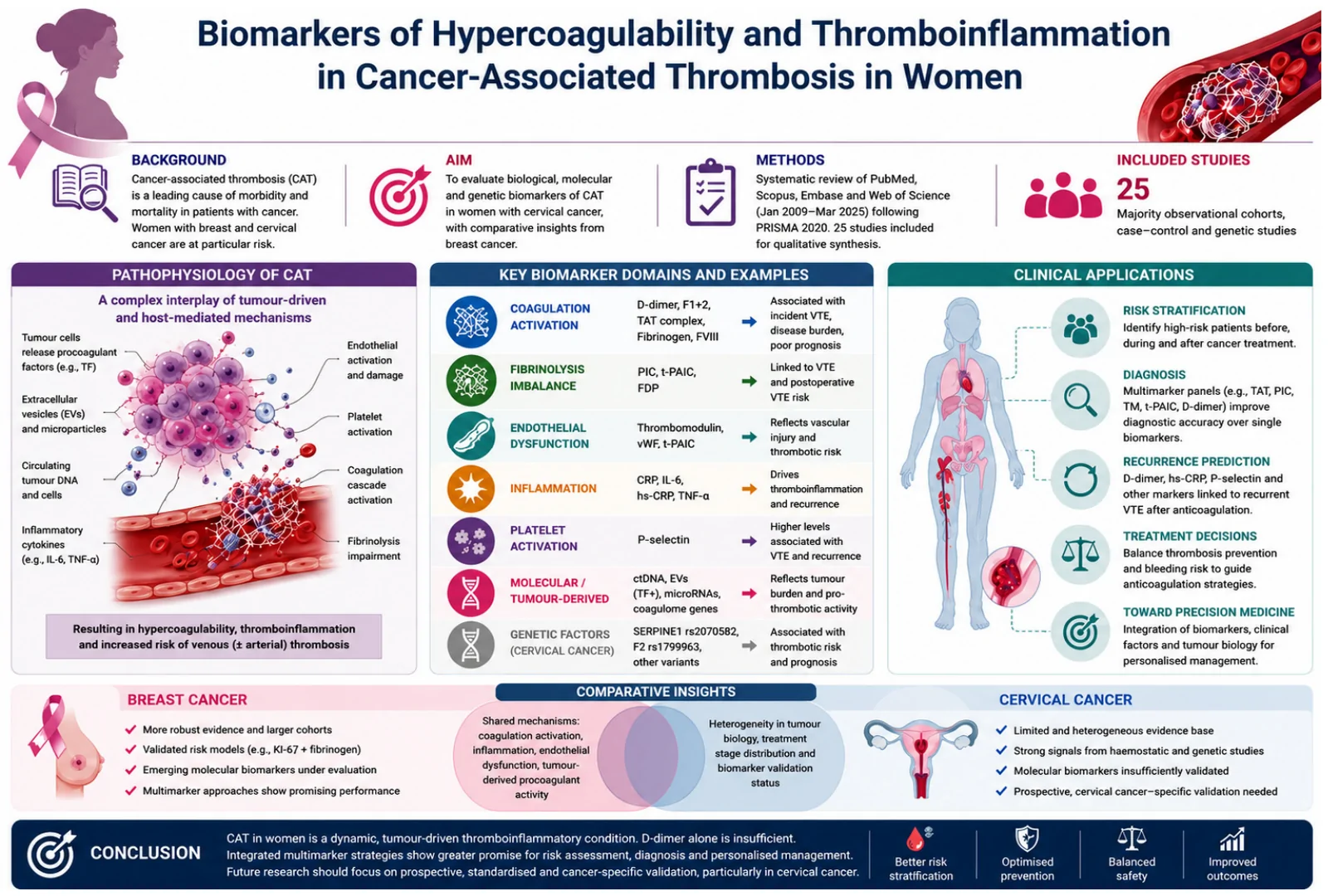
Integrated biomarker framework of cancer-associated thrombosis in women with breast and cervical cancer. The figure summarises the principal biological mechanisms, biomarker domains, clinical applications, and comparative translational insights identified in this systematic review.

These comparative findings support the development of cancer type-specific approaches to thrombosis risk assessment rather than universal biomarker models. The integrated biological domains, clinical applications, and comparative translational framework are summarised in Figure 2 and Table 1, Table 2, Table 3 and Table 4.

## 4. Discussion

This systematic review synthesises evidence from 25 cervical cancer studies and selected translational breast cancer data to provide a comprehensive, biologically informed perspective on biomarkers of hypercoagulability and thromboinflammation in CAT. The principal finding of this review is that CAT in women is a biologically heterogeneous and dynamically evolving thromboinflammatory syndrome, driven by tumour-specific mechanisms that extend well beyond the prothrombotic changes captured by conventional biomarkers such as D-dimer. This complexity supports the development of integrated, multimodal, and cancer-adapted precision approaches rather than reliance on single biomarkers [9,47,48,54].

### 4.1. D-Dimer: Clinically Valuable but Insufficient in Isolation

D-dimer remains the most extensively investigated and clinically accessible biomarker for CAT because of its high sensitivity for fibrin formation and degradation. However, this review highlights a fundamental limitation in oncology settings: elevated D-dimer concentrations frequently reflect tumour burden, systemic inflammation, endothelial activation, and treatment-related vascular injury in the absence of objectively confirmed thrombosis. Specificity is thereby substantially compromised, and reliance on isolated D-dimer assessment risks both under-identification and over-investigation in cancer populations [18,21,47,53,54,55].

These findings support the use of D-dimer as a screening or baseline risk stratification tool within multimarker frameworks rather than as a standalone diagnostic or predictive test. Integration with biomarkers reflecting active haemostatic dysregulation, endothelial injury, and inflammatory activation may improve discriminatory performance [9,21,47,54].

### 4.2. Active Haemostatic and Fibrinolytic Dysregulation

Biomarkers directly reflecting thrombin generation—TAT complexes and F1+2—and fibrinolytic activation, particularly PIC, demonstrated greater mechanistic specificity for ongoing coagulation dysregulation than D-dimer alone [19,21,23,48]. In cervical cancer, PIC showed promising perioperative predictive value, suggesting an important role of fibrinolytic dysregulation in tumour-associated hypercoagulability that has been comparatively underexplored [23].

The findings support TAT, F1+2, and PIC as candidate components of multimarker haemostatic profiling for CAT risk stratification and diagnosis. However, assay standardisation, reference ranges in cancer populations, and cut-off validation across different clinical contexts remain important unresolved challenges [19,21,48].

### 4.3. Thromboinflammation and Vascular Dysfunction

The consistent co-elevation of inflammatory biomarkers (CRP, hs-CRP, IL-6) with coagulation and fibrinolysis markers across included studies supports the central role of thromboinflammation in CAT pathogenesis. These findings support a model in which tumour-driven cytokine release, NETosis, and immunothrombotic signalling amplify haemostatic activation synergistically with direct procoagulant tumour activity [9,20].

Endothelial dysfunction—evidenced by elevated TM and t-PAIC—and platelet activation—reflected by P-selectin—further contribute to vascular homeostasis disruption in CAT [21]. P-selectin demonstrated utility for recurrence-risk assessment even after initiation of anticoagulant therapy [9,52], suggesting a role in identifying patients with persistent biological thrombotic vulnerability [52].

### 4.4. Molecular and Tumour-Derived Biomarkers: Toward Precision Profiling

Emerging molecular biomarkers—ctDNA, TF-positive EVs, and microRNAs—represent promising candidates for future precision thrombosis medicine in oncology. They offer the capacity to capture dynamic tumour biology, procoagulant signalling, and systemic thromboinflammatory activation in ways that conventional haemostatic biomarkers cannot [9,10,12,13,14,15,16,17]. ctDNA showed associations with VTE risk in pan-cancer cohorts [10]; TF-positive EVs demonstrated strong mechanistic and emerging clinical evidence for their role in CAT [12,13,14,15,17]; and microRNAs may provide insights into post-transcriptional modulation of thromboinflammatory pathways [16,26].

Nevertheless, these biomarkers currently lack prospective, tumour-specific clinical validation, face significant analytical heterogeneity (particularly EVs), and require standardised assay methodologies before clinical implementation becomes feasible. Integration with haemostatic and inflammatory biomarkers within multimodal frameworks is likely to facilitate clinical translation [9,21,54].

For a molecular biomarker to be considered a shared translational marker of CAT across cervical and breast cancer, future studies would need to demonstrate comparable expression patterns within similar disease stages and show that its association with VTE remains independent of tumour type, tumour burden, treatment exposure, and systemic inflammation.

### 4.5. Genetic Determinants and Tumour Coagulome Signatures

Genetic variants in SERPINE1 and F2 were associated with thrombotic susceptibility and adverse prognosis in cervical cancer cohorts [24,25], providing biological support for the concept that tumour-specific genetic reprogramming of the coagulome may contribute to cancer-induced hypercoagulability. These findings suggest that genomic profiling may complement haemostatic and molecular biomarker assessment in future precision thrombosis risk models [9,24,25]. However, the available data derive from small exploratory cohorts, and clinical application requires validation in prospective, adequately powered studies.

### 4.6. Proposed Precision Biomarker Framework

To facilitate the future development of biomarker-guided thrombosis management, Table 4 presents a proposed precision biomarker framework for CAT in women that integrates current evidence on biological role, clinical application, evidence maturity, translational potential, and key limitations across biomarker categories. The framework is intended as a structured translational reference to support future research and biomarker development rather than as a validated clinical decision-making tool. Prospective tumour-specific studies are required to validate its clinical applicability before routine implementation.

### 4.7. Clinical Implications

The findings of this review have several direct clinical implications for precision CAT management. First, the limited specificity of D-dimer in isolation supports a transition toward integrated multimarker strategies combining coagulation, inflammatory, endothelial, and tumour-derived biomarkers to improve identification of patients with persistent hypercoagulability. Second, dynamic longitudinal biomarker monitoring may facilitate more individualised thromboprophylaxis and anticoagulation management decisions throughout disease progression and treatment exposure. Third, the comparative analysis between breast and cervical cancer indicates that biomarker-guided thromboprophylaxis will likely require tumour-type adaptation rather than universal implementation.

Integration of molecular biomarker profiling—including ctDNA, EVs, and coagulome-associated signatures—into clinical oncology workflows may eventually support more personalised thrombosis risk assessment. However, such approaches remain investigational and require prospective tumour-specific validation before they can inform thromboprophylaxis decisions, anticoagulation duration, bleeding-risk mitigation, or routine clinical management in women with CAT.

### 4.8. Strengths and Limitations

The principal strengths of this review are: (i) systematic integration of evidence across haemostatic, inflammatory, endothelial, molecular, and genetic biomarker domains; (ii) a structured comparative translational framework between breast and cervical cancer providing biologically informed, tumour-specific perspectives on CAT; (iii) incorporation of emerging concepts including thromboinflammation, EVs, tumour coagulome signalling, and precision thrombosis profiling; and (iv) focus on underrepresented female malignancies, particularly cervical cancer, with explicit identification of major evidence gaps.

Several limitations must be acknowledged. The available clinical evidence was highly heterogeneous regarding cancer population, disease stage, treatment exposure, biomarker assay, sample timing, cut-off definitions, and outcome definitions, precluding quantitative meta-analysis and limiting direct biomarker performance comparisons. The evidence base was uneven between tumour types: breast cancer and mixed-cancer cohorts provided a broader clinical and translational framework, whereas cervical cancer-specific evidence remained limited and predominantly exploratory.In addition, most cervical cancer studies included in this review originated from East Asian populations, which may limit the generalizability of the findings to other geographic regions with different patient characteristics, healthcare systems, and HPV genotype distributions. The review protocol was not prospectively registered in PROSPERO.

These findings should therefore be regarded as a structured synthesis of current clinical and translational evidence and as a framework for future prospective validation, rather than as immediate justification for biomarker-guided clinical implementation.

Another important limitation concerns disease-stage heterogeneity. The included studies evaluated patients across different phases of cervical cancer progression, ranging from operable or localised disease to locally advanced and metastatic settings. These stages are not biologically equivalent. Metastatic disease is associated with increased tumour burden, altered secretome composition, extracellular vesicle release, ctDNA shedding, microRNA dysregulation, angiogenic activation, endothelial injury, systemic inflammation, and coagulation activation, all of which may influence both biomarker levels and thrombotic risk. Therefore, aggregation across tumour stages may dilute stage-specific vascular or molecular signatures and limit the ability to identify biomarkers associated with CAT independently of tumour progression. Future studies should prospectively evaluate biomarker performance according to disease stage, metastatic status, treatment exposure, and tumour burden.

Another limitation relates to the geographic and methodological profile of the available evidence. Most cervical cancer studies included in this review were observational and originated predominantly from Asian and European populations. This may limit generalisability, particularly because cervical cancer biology is influenced by geographic variation in HPV genotype distribution, access to screening, stage at diagnosis, treatment patterns, and population-specific inflammatory or genetic backgrounds.High-risk HPV genotypes, including HPV16 and HPV18, differ in oncogenic potential and may differentially influence immune evasion, angiogenesis, endothelial activation, and tumour-associated inflammatory signalling. Therefore, differences in HPV-related molecular drivers across regions may influence biomarker expression and thromboinflammatory phenotypes. In addition, the observational nature of most included studies limits causal inference and prevents definitive conclusions regarding whether the reported biomarkers directly contribute to thrombosis, reflect tumour burden or inflammation, or act as surrogate markers of disease severity.

### 4.9. Future Directions

Future research should prioritise prospective, multicentre, longitudinal studies evaluating validated multimarker panels in cancer type-specific populations, including cervical cancer. Moreover, there remains a complete absence of prospective biomarker-guided interventional studies evaluating whether biomarker-informed thromboprophylaxis or anticoagulation strategies improve clinical outcomes in women with cervical cancer. Standardisation of biomarker assay methodologies—particularly for EVs and thrombin generation testing—across clinical sites is a prerequisite for meaningful cross-study comparisons.

Integration of molecular tumour profiling (coagulome signatures, ctDNA trajectories, EV phenotyping) with haemostatic and inflammatory biomarkers may enable biologically informed precision thrombosis models. Machine learning and artificial intelligence-assisted analytical approaches applied to multimodal longitudinal biomarker data may further improve prediction of thrombotic recurrence, bleeding risk, and treatment-related vascular toxicity beyond conventional statistical frameworks. Future precision oncology strategies are expected to integrate tumour biology, thromboinflammation, vascular dysfunction, and multimodal biomarker profiling into dynamic, personalised approaches to thrombosis prevention and anticoagulation management.

## 5. Conclusions

This systematic review highlights that CAT in women represents a biologically complex and dynamically evolving thromboinflammatory syndrome driven by tumour-specific mechanisms, systemic inflammation, endothelial dysfunction, platelet activation, and haemostatic dysregulation. The findings support a paradigm shift from conventional "one-size-fits-all" thrombosis models toward precision medicine approaches grounded in tumour-specific hypercoagulability and integrated biological profiling.

While D-dimer remains clinically valuable because of its sensitivity and accessibility, its limited specificity in oncology populations underscores the need for multidimensional biomarker strategies integrating coagulation activation, fibrinolysis, inflammation, endothelial dysfunction, and tumour-derived molecular signalling. Emerging biomarkers—including ctDNA, EVs, microRNAs, and coagulome-related pathways—offer promising opportunities to better characterise tumour-associated hypercoagulability and support more biologically informed thrombosis risk assessment.

The comparative analysis between breast and cervical cancer highlights both shared thromboinflammatory pathways and tumour-specific differences influencing biomarker performance. While breast cancer benefits from more advanced translational biomarker development, cervical cancer remains substantially underrepresented in precision thrombosis research, representing a critical unmet need in women’s oncology.

Despite growing translational evidence, the current literature remains heterogeneous and insufficiently standardised, particularly regarding longitudinal monitoring, recurrence-risk prediction, bleeding-risk assessment, and biomarker-guided anticoagulation. Future research should prioritise prospective, tumour-specific, multimodal studies integrating haemostatic, inflammatory, molecular, and computational biomarkers within dynamic precision medicine frameworks, with the goal of supporting validated and clinically applicable approaches to thrombosis prevention and management across the continuum of cancer progression and treatment.

## Figures and Tables

**Table 1:** Overview of biomarkers of hypercoagulability and thromboinflammation in cervical cancer and their reported clinical significance.

Study (Reference)	Country	Study Design	Sample Size (n)	Cancer Stage	Biomarkers/Risk Factors	Outcome	Main Findings
Liang et al., 2020 [18]	China	Retrospective observational study	134	I–IV	D-dimer, fibrinogen, platelet count, PT, APTT, TT		Elevated D-dimer, fibrinogen, and platelet count were associated with an increased risk of VTE; combined biomarker assessment improved identification of high-risk patients.
Qiu et al., 2024 [19]	China	Retrospective cohort	177	I–IV	TAT, PIC, TM, t-PAIC, D-dimer, FDP	VTE	Elevated TAT predicted VTE; combined thrombotic biomarkers improved diagnostic accuracy.
Li et al., 2023 [20]	China	Retrospective cohort study	795	IB–IVA	Fibrinogen, PLR, NLR, MLR, SIRI, AISI	Overall survival	Elevated pretreatment fibrinogen and inflammatory indices were independently associated with poorer overall survival in patients with advanced cervical cancer.
Neto et al., 2024 [24]	Portugal	Retrospective cohort	401	Not clearly reported	SERPINE1 rs2070682, F2 rs1799963 and other haemostatic SNPs	VTE and overall survival	SERPINE1 rs2070682 independently predicted cervical cancer-associated VTE; F2 rs1799963 was associated with poorer survival.
Neto et al., 2023 [25]	Portugal	Retrospective hospital-based cohort	400	I–IV	PROCR rs10747514, RGS7 rs2502448 and six other thrombogenesis-related SNPs; Khorana score	Cervical cancer-associated VTE and overall survival	PROCR and RGS7 variants independently predicted VTE risk; the Khorana score showed poor performance.
Costa et al., 2025 [26]	Portugal	Case-control study	137	I–IV	Plasma microRNAs	Cancer-associated thrombosis	Plasma microRNAs showed potential as biomarkers of cancer-associated thrombosis in cervical cancer.
Matsuo et al., 2016 [28]	USA	Retrospective cohort	798	I–IV	Clinical and tumour-related VTE risk factors; albumin	VTE; overall survival	VTE was associated with aggressive tumour characteristics and was an independent prognostic factor for decreased survival
Zhao et al., 2022 [29]	China	Retrospective cohort	338	I–IV	D-dimer	Perioperative VTE	High D-dimer levels were independently associated with preoperative VTE in patients undergoing surgery for cervical cancer
Li et al., 2019 [30]	China	Prospective observational cohort	198	Not specified	D-dimer, PDW	Postoperative DVT	Elevated preoperative D-dimer independently predicted postoperative DVT; combining D-dimer with PDW improved risk prediction.
Zhao et al., 2015 [31]	China	Retrospective cohort	220	I–IIA	Pretreatment plasma fibrinogen, platelet count	Recurrence and survival	Hyperfibrinogenaemia independently predicted poorer survival; elevated fibrinogen and platelet levels were associated with recurrence.
Zayyan et al., 2019 [32]	Nigeria	Prospective cross-sectional study	67	I–IVB	D-dimer, Wells score	D-dimer, Wells score	DVT occurred only in patients with elevated D-dimer and a high Wells score.
Tasaka et al., 2020 [33]	Japan	Retrospective cohort	754	I–IV	Pre-treatment D-dimer	Pretreatment VTE	Pretreatment VTE occurred in 7.3%; older age and larger tumours were independent risk factors.
Tietie et al., 2023 [34]	Nigeria	Prospective case-control study	65	I–IV	Plasma D-dimer	Disease stage/hypercoagulability	Higher D-dimer levels were associated with advanced-stage cervical cancer.
Cheng et al., 2024 [35]	China	Retrospective cohort	309	Not clearly reported	Preoperative D-dimer, age, obesity, triglycerides, surgical approach	Postoperative lower-extremity DVT	Elevated D-dimer and clinical factors independently predicted postoperative DVT.
Chen et al., 2024 [36]	China	Retrospective cohort	888	Early-stage cervical cancer	Plasma D-dimer	Recurrence/hypercoagulability	Elevated preoperative D-dimer was independently associated with increased recurrence risk.
Wang et al., 2020 [37]	China	Comparative clinical cohort	60	Not clearly reported	miR-195-5p, miR-205-5p, VEGF-A	VTE and treatment response	Lower miR-195-5p and miR-205-5p and higher VEGF-A were associated with VTE.
Polterauer et al., 2009 [38]	Austria	Retrospective cohort	158	IB–IV	Plasma fibrinogen	Survival/hypercoagulability	Elevated preoperative fibrinogen independently predicted poor prognosis.
Chen et al., 2024 [39]	China	Retrospective cohort with internal validation	1174	I–IV	D-dimer, FDP, NLR, age, histological type, chemotherapy	Postoperative VTE	D-dimer, FDP, and NLR independently predicted postoperative VTE.
Tsai et al., 2012 [40]	Taiwan	Nationwide population-based retrospective cohort	1013	Not reported	Age, hypertension, diabetes, coronary artery disease, hyperlipidaemia, treatment modality	VTE incidence and survival	Cervical cancer was associated with a tenfold higher VTE risk; VTE was associated with poorer survival.
Yuk et al., 2021 [41]	South Korea	Nationwide retrospective cohort	49,514	Not reported	Age, comorbidity burden, surgery, radiotherapy, chemotherapy	VTE incidence and risk	Chemotherapy was associated with the highest VTE risk, followed by radiotherapy and surgery.
Piškur et al., 2023 [42]	Croatia	Retrospective observational study	66	IA–IVB	VEGF-A	Disease progression	High VEGF-A expression was associated with more aggressive disease and poorer prognosis, supporting VEGF-A as a marker of angiogenesis and vascular activation in cervical cancer.
Zhao et al., 2018 [43]	China	Retrospective observational study	258	I–IV	Tissue factor (TF)	Lymph node metastasis, disease progression	High TF expression was associated with advanced disease, supporting TF as a marker of tumor-associated hypercoagulability.
Slukhanchuk et al., 2023 [44]	Russia	Cross-sectional comparative observational study	73 cancer patients; cervical cancer subgroup n = 9	I–III	citH3, MPO:Ag, vWF:Ag, ADAMTS-13 antigen/activity, D-dimer	Immunothrombosis and endothelial activation	In cervical cancer, citH3 correlated strongly with vWF:Ag and MPO:Ag, indicating NET-associated endothelial activation.
Domenici et al., 2021 [45]	Italy	Observational prognostic cohort study	108	Mixed stages	Platelet count, PLR, IL-6	DFS, OS	Higher platelet count, PLR, and IL-6 expression were associated with poorer survival.
Nakamura et al., 2018 [46]	Japan	Retrospective single-center cohort study	98	I–IV	NLR, PLR, platelet count, D-dimer, fibrinogen	PFS, OS	High pretreatment NLR independently predicted poorer progression-free and overall survival.

**Table 2:** Established and emerging biomarkers for the prediction and characterisation of cancer-associated thrombosis (CAT). Biomarkers are grouped according to the principal pathophysiological mechanisms involved in CAT. Evidence level refers to the maturity of biomarker evidence for CAT-related clinical interpretation, considering consistency across studies, tumour-specific validation, reproducibility, assay standardisation, and proximity to clinical implementation. It does not represent a formal GRADE assessment or validated predictive accuracy for VTE.

Domain	Biomarker	Biological Function	Association with CAT	Potential Clinical Application	Evidence	Main References
Coagulation Activation	D-dimer	Marker of fibrin formation and degradation	Consistently associated with increased CAT risk, disease progression, and mortality; limited specificity	Baseline risk assessment, prognostic stratification, treatment monitoring	High	[18,23,24,47]
	F 1+2	Marker of in vivo thrombin generation	Associated with hypercoagulability and increased VTE risk	Global coagulation activation profiling and risk prediction	Moderate	[48]
	TAT Complex	Marker of active thrombin formation	Reflects ongoing coagulation activation and thrombotic tendency	Component of multimarker thrombosisrisk models	Moderate	[19,21]
Fibrinolytic Activity	Plasmin–α2 Plasmin Inhibitor Complex	Marker of plasmin generation and fibrinolytic activity	Associated with VTE occurrence and postoperative thrombotic complications	Perioperative and longitudinal thrombosis risk assessment	Moderate	[19,21,23]
Endothelial Activation/Dysfunction	Thrombomodulin (TM), t-PAIC	Markers of endothelial injury and dysfunction	Associated with endothelial perturbation and thrombotic complications	Assessment of vascular injury and thrombo-inflammatory status	Moderate	[19,21]
Thromboinflammation	CRP, hs-CRP, IL-6	Markers of systemic inflammation and immune activation	Associated with incident and recurrent VTE and adverse outcomes	Prognostic assessment and recurrence risk stratification	Moderate	[45]
Platelet Activation	P-selectin	Marker of platelet and endothelial activation	Associated with VTE occurrence and recurrence; incorporated into risk prediction models	Risk stratification and refinement of CAT prediction scores	Moderate–High	[9]
Tumour-Derived Molecular Biomarkers	Circulating Tumour DNA (ctDNA)	Reflects tumour burden, genomic alterations, and biological aggressiveness	Emerging evidence links ctDNA burden with thrombotic risk	Dynamic risk assessment and disease monitoring	Emerging	[10]
	microRNAs	Post-transcriptional regulators of coagulation, inflammation, and tumour progression	Exploratory associations with CAT susceptibility and prognosis	Precision thrombosis phenotyping and biomarker panels	Emerging	[16,26]
Extracellular Vesicles	TF-positive EVs/MPs	Tumour-derived carriers of tissue factor with potent procoagulant activity	Strong mechanistic association with CAT development and progression	Translational biomarker for thrombosis risk and therapeutic targeting	Moderate	[12,13,14,15,17]
Genetic and Genomic Biomarkers	SERPINE1, F2 and related variants	Regulation of coagulation and fibrinolytic pathways	Associated with thrombotic susceptibility and clinical outcomes	Genetic risk assessment and precision-medicine approaches	Moderate	[24,25]

**Table 3:** Comparative evidence supporting biomarkers of cancer-associated thrombosis (CAT) in cervical and breast cancer. Biomarkers are compared according to the current evidence base in cervical and breast cancer, biological relevance, clinical utility, and translational potential. D-dimer remains the most extensively validated biomarker in both malignancies, whereas biomarkers reflecting thrombin generation, fibrinolysis, endothelial dysfunction, platelet activation, tumour biology, extracellular vesicle (EV) activity, and genetic susceptibility represent emerging approaches for precision thrombosis risk assessment. Collectively, these findings support the transition from isolated biomarker evaluation toward integrated multimodal models incorporating clinical, biological, molecular, and tumour-specific determinants of thrombotic risk. * The evidence categories presented in this framework reflect the current translational maturity and clinical readiness of each biomarker rather than formal evidence grading or validated predictive accuracy for VTE. Biomarkers with strong mechanistic plausibility, such as ctDNA, microRNAs, and TF-positive extracellular vesicles, should therefore be interpreted as emerging or exploratory tools requiring tumour-specific, stage-specific, and prospective clinical validation.

Biomarker (References)	Evidence * in Cervical Cancer	Evidence * in Breast Cancer	Clinical Relevance	Translational Perspective
D-dimer [18,29,30,32,36,39,47,51,54]	Consistently associated with VTE occurrence; predictive value demonstrated across multiple cohorts, although specificity remains limited	Associated with VTE, disease progression, and adverse clinical outcomes	Risk assessment and prognostic stratification	Best-validated biomarker; performance may improve when incorporated into multimarker models
Thrombin–antithrombin (TAT) complex [19,21]	Limited but supportive evidence as a marker of coagulation activation	Reflects increased thrombin generation and hypercoagulability	Assessment of active coagulation	Dynamic biomarker suitable for integrated haemostatic profiling
Plasmin–α2 plasmin inhibitor complex (PIC) [21,23]	Associated with VTE risk, particularly in perioperative settings	Limited direct evidence available	Assessment of fibrinolytic activation	Promising biomarker for perioperative thrombosis risk stratification
Thrombomodulin (TM)/t-PAIC [19,21]	Markers of endothelial injury associated with thrombotic complications	Evidence supports a role for endothelial dysfunction in thrombosis pathogenesis	Evaluation of vascular injury and endothelial activation	Potential components of endothelial biomarker panels
CRP/IL-6 [9,45]	Associated with advanced disease, systemic inflammation, and thrombotic risk	Linked to VTE, recurrence, and poor clinical outcomes	Assessment of thromboinflammatory burden	Candidate biomarkers for inflammation-based risk prediction models
P-selectin [9]	Limited cervical cancer-specific evidence	Associated with platelet activation, VTE occurrence, and recurrence risk	Marker of platelet activation	May identify residual thrombotic risk and complement prediction models
Circulating tumour DNA (ctDNA) [10]	Preliminary evidence; limited disease-specific validation	Emerging evidence links ctDNA burden to VTE risk across cancer types	Surrogate marker of tumour burden and biological aggressiveness	Dynamic precision biomarker integrating tumour evolution and thrombosis risk
TF-positive extracellular vesicles/microparticles [12,13,14,15,17]	Limited direct clinical validation	Strong biological and mechanistic evidence supporting a role in CAT	Marker of tumour-driven coagulation activation	Promising tool for molecular thrombosis phenotyping and therapeutic targeting
SERPINE1, F2 and related genetic variants [24,25]	Genetic associations reported with thrombosis susceptibility	Limited breast cancer-specific validation	Assessment of inherited and tumour-associated thrombotic predisposition	Supports the development of precision thrombosis models incorporating genomic data

**Table 4:** Proposed precision biomarker framework for risk stratification of cancer-associated thrombosis (CAT) in women with cancer. Biomarkers are categorised according to their principal biological roles, current clinical applications, evidence maturity, translational potential, and key limitations. Conventional biomarkers, including D-dimer, thrombin generation markers, fibrinolytic biomarkers, and inflammatory markers, remain clinically relevant for baseline risk assessment and disease monitoring but have limited specificity when used individually. Emerging molecular biomarkers, including circulating tumour DNA (ctDNA), extracellular vesicles (EVs), microRNAs, and tumour-associated genetic variants, offer promising opportunities for precision thrombosis risk assessment but require further prospective, tumour-specific validation. Future biomarker-guided approaches are expected to integrate clinical variables with haemostatic, inflammatory, molecular, and genomic biomarkers to enable personalised thrombosis prevention and management.

Biomarker	Biological Role	Clinical Application	Evidence Maturity	Translational Potential	Key Limitation
D-dimer	Fibrin formation/degradation	Screening; baseline risk assessment	High	Moderate	Low specificity in oncology; reflects non-thrombotic processes
TAT complex/F1+2	Thrombin generation markers	Hypercoagulability profiling; multimarker panels	Moderate	High	Limited assay standardisation
PIC	Fibrinolytic activation	Perioperative and longitudinal VTE risk assessment	Moderate	High	Limited prospective validation
CRP/IL-6/hs-CRP	Systemic thromboinflammation	Recurrence-risk and prognostic stratification	Moderate	Moderate	Non-specific for thrombosis
P-selectin	Platelet and endothelial activation	Residual thrombotic risk; recurrence prediction	Moderate–High	High	Limited tumour-specific validation
ctDNA	Tumour burden; genomic instability	Dynamic precision risk assessment; treatment monitoring	Emerging	Very high	No prospective thrombosis-specific validation
TF-positive EVs/MPs	Tumour-derived procoagulant activity	Molecular thrombosis phenotyping	Moderate	Very high	Analytical and methodological heterogeneity
microRNAs	Post-transcriptional coagulation/inflammation regulation	Precision biomarker discovery	Emerging	High	Predominantly exploratory evidence
SERPINE1/F2 variants	Genetic coagulation–fibrinolysis balance	Genetic susceptibility assessment; precision models	Moderate	Moderate	Exploratory cohorts; requires prospective validation

## Data Availability

Data are available within the article and Supplementary Materials.

## Supplementary Materials

The following supporting information can be downloaded at https://www.mdpi.com/article/10.3390/ijms27167302/s1.

## Abbreviations

ATE	Arterial thrombotic event
CAT	Cancer-associated thrombosis
CRP	C-reactive protein
CTCs	Circulating tumour cells
ctDNA	Circulating tumour DNA
DVT	Deep vein thrombosis
EVs	Extracellular vesicles
F1+2	Prothrombin fragment 1+2
hs-CRP	High-sensitivity C-reactive protein
IL-6	Interleukin-6
MPs	Microparticles
NETs	Neutrophil extracellular traps
NOS	Newcastle–Ottawa Scale
PE	Pulmonary embolism
PIC	Plasmin–α2-antiplasmin complex
PRISMA	Preferred Reporting Items for Systematic Reviews and Meta-Analyses
TAT	Thrombin–antithrombin complex
TF	Tissue factor
TM	Thrombomodulin
t-PAIC	Tissue plasminogen activator inhibitor complex
VTE	Venous thromboembolism
