# Supplementary material for: Biomarkers of Hypercoagulability and Thromboinflammation in Cervical Cancer-Associated Thrombosis: A Systematic Review with Translational Insights from Breast Cancer
Source: Int J Mol Sci. 2026 Aug 16;27(16):7302. doi: 10.3390/ijms27167302 (PMC13513456; doi:10.3390/ijms27167302)
Supplement: Supplementary file 1 [file ijms-27-07302-s001.zip › ijms-4392513-supplementary.pdf]

Supplementary Table S1. Detailed Database Search Strategies

Database	Search Strategy
PubMed/MEDLINE	("cervical cancer" OR "cervical neoplasm" OR "cervical carcinoma") AND ("cancer-associated thrombosis" OR "venous thromboembolism" OR "deep vein thrombosis" OR "hypercoagulability" OR "thromboinflammation") AND ("biomarker" OR "D-dimer" OR "thrombin generation" OR "thrombin-antithrombin complex" OR "plasmin-antiplasmin complex" OR "extracellular vesicles" OR "tissue factor" OR "circulating tumor DNA" OR "microRNA" OR "coagulome")
Embase	('cervical cancer'/exp OR 'cervical carcinoma') AND ('cancer associated thrombosis' OR 'venous thromboembolism' OR 'hypercoagulability' OR 'thromboinflammation') AND ('biomarker' OR 'D-dimer' OR 'thrombin generation' OR 'extracellular vesicle' OR 'tissue factor' OR 'circulating tumor DNA' OR 'microRNA')
Scopus	TITLE-ABS-KEY ("cervical cancer" AND "cancer-associated thrombosis" AND biomarkers AND hypercoagulability) OR TITLE-ABS-KEY ("cervical cancer" AND thromboinflammation AND venous thromboembolism)
Web of Science	TS=("cervical cancer" AND "venous thromboembolism" AND biomarkers AND thromboinflammation) OR TS=("cervical neoplasm" AND hypercoagulability AND coagulation biomarkers)

Additional Search Information
Search period: January 2009 – March 2025
Final search conducted: March 2025
Languages: English and Russian
Additional manual searches were performed through reference list screening of eligible studies and relevant review articles.

Supplementary Table S2. Detailed Eligibility Criteria

Domain	Inclusion Criteria	Exclusion Criteria
Population	Adult women with cervical cancer	Paediatric populations, male-only cohorts, non-malignant diseases
Cancer type	Cervical cancer; breast cancer studies included only for translational comparison	Studies without extractable cervical cancer-related information
Study design	Prospective cohorts, retrospective cohorts, case–control studies, translational biomarker studies, genetic association studies	Narrative reviews, systematic reviews, meta-analyses, editorials, conference abstracts, case reports
Biomarkers	Biological, haemostatic, inflammatory, endothelial, molecular, and genetic biomarkers	Studies without biomarker assessment
Outcomes	VTE, DVT, PE, recurrent thrombosis, thrombosis-related complications, thrombosis-associated prognosis	Studies without thrombosis-related outcomes
Publication type	Peer-reviewed full-text studies	Non-peer-reviewed publications
Language	English or Russian	Other languages

Study setting	Ambulatory, perioperative, inpatient, outpatient, palliative, chemotherapy-associated settings	Non-clinical experimental-only studies
Mechanistic studies	Included only for contextual translational interpretation	Not included in primary systematic synthesis

SupplementaryTable S3. Methodological quality assessment of studies included in the review. Clinical observational studies were assessed using the Newcastle–Ottawa Scale (NOS), which evaluates study quality according to participant selection, comparability of study groups, and outcome ascertainment. Studies scoring 7–9 points were considered high quality, 5–6 points moderate quality, and <5 points low quality. Purely mechanistic, non-clinical translational, and review-based studies were not amenable to formal NOS assessment and are therefore presented as descriptive supporting evidence. Cross-sectional studies were not assigned a standard NOS score because the original Newcastle–Ottawa Scale was developed for cohort and case–control studies. Overall, the available clinical evidence was predominantly of moderate-to-high methodological quality, although limitations related to observational study design, cancer heterogeneity, small sample size, restricted disease-specific analyses, and limited external validation were frequently identified.

Study (Reference)	Study Design	NOS Score (0–9)	Methodological Quality	Principal Limitation
Liang et al. [18]	Retrospective observational study	8/9	High	Retrospective single-centre design
Qiu et al. [19]	Retrospective cohort	7/9	High	Retrospective design and limited external validation
Li et al. [20]	Retrospective cohort study	8/9	High	Survival rather than VTE as the primary endpoint
Neto et al. [24]	Retrospective cohort	8/9	High	Exploratory genetic analysis; limited external validation
Neto et al. [25]	Retrospective hospital-based cohort	8/9	High	Hospital-based retrospective design
Costa et al. [26]	Case–control study	6/9	Moderate	Exploratory molecular biomarker study requiring

				external validation
Matsuo et al. [29]	Retrospective cohort	8/9	High	Retrospective design and potential residual confounding
Zhao et al. [30]	Retrospective cohort	8/9	High	Perioperative population and retrospective design
Li et al. [31]	Prospective observational cohort	8/9	High	Limited external validation
Zhao et al. [32]	Retrospective cohort	7/9	High	Early-stage surgical population
Zayyan et al. [33]	Prospective cross-sectional study	N/A	Descriptive evidence	Cross-sectional design and small sample size
Tasaka et al. [34]	Retrospective cohort	8/9	High	Mixed gynaecological cancer population
Tietie et al. [35]	Prospective case–control study	6/9	Moderate	Small single-centre cohort
Cheng et al. [36]	Retrospective cohort	8/9	High	Retrospective perioperative study
Chen et al. [37]	Retrospective cohort	8/9	High	Early-stage surgical population
Wang et al. [38]	Comparative clinical cohort	6/9	Moderate	Small sample size

Polterauer et al. [39]	Retrospective cohort	7/9	High	Single-centre retrospective study
Chen et al. [40]	Retrospective cohort with internal validation	8/9	High	External validation was not performed
Tsai et al. [41]	Nationwide population-based retrospective cohort	8/9	High	Administrative database; limited biomarker data
Yuk et al. [42]	Nationwide retrospective cohort	8/9	High	Claims database; no laboratory biomarkers
Piškur et al. [43]	Retrospective observational study	6/9	Moderate	Small single-institution cohort
Zhao et al. [44]	Retrospective observational study	8/9	High	Tissue-based retrospective analysis
Slukhanchuk et al. [45]	Cross-sectional comparative observational study	N/A	Descriptive evidence	Very small cervical cancer subgroup (n = 9)
Domenici et al. [46]	Observational prognostic cohort study	6/9	Moderate	Small cohort and prognostic design
Nakamura et al. [47]	Retrospective single-centre cohort study	6/9	Moderate	Single-centre retrospective study

Footnote. NOS scoring was applied only to eligible observational clinical studies. Mechanistic, translational, and review-based studies were not evaluated using NOS because of methodological incompatibility. Cross-sectional studies were not assigned a standard NOS score because the original Newcastle–Ottawa Scale was developed for cohort and case–control studies.

Abbreviations: NOS, Newcastle–Ottawa Scale.

Supplementary Table S4. Mechanistic and Translational Studies Included for Contextual Interpretation

Study	Study Type	Translational/Mechanistic Contribution
Falanga et al. [8]	Mechanistic review	Coagulation activation and tumour biology
Hisada and Mackman [12]	Translational review	Tissue factor-positive extracellular vesicles and thrombosis
Zwicker et al. [19]	Prospective biomarker study	Tumour-derived microparticles associated with VTE
Tesselaar et al. [13]	Biomarker/mechanistic study	Microparticle tissue factor activity
Labelle et al. [11]	Experimental mechanistic study	Platelet–tumour interaction and metastasis
Ma et al. [10]	Prospective molecular biomarker study	ctDNA and thrombosis prediction
Gardiner et al. [14]	Mechanistic review	Extracellular vesicles and cancer thrombosis
Beck et al. [15]	Mechanistic review	Inflammation–cancer–thrombosis interactions
Kim et al. [9]	Translational review	Biomarkers and mechanisms of CAT
Giaccherini et al. [33]	Translational biomarker study	Thrombotic biomarkers and breast cancer prognosis

Footnote. These studies were not included in the primary systematic synthesis of cervical cancer biomarker studies but were incorporated to provide comparative translational insights and mechanistic context for cancer-associated hypercoagulability.

Supplementary Figure S1. PRISMA 2020 Checklist

Section/Topic	PRISMA 2020 Item	Reported in Manuscript
Title	Identify the report as a systematic review	Title page
Abstract	Structured summary	Abstract
Introduction	Rationale and objectives	Sections 1.1–1.7
Methods	Eligibility criteria	Section 2.3
Methods	Information sources	Section 2.2
Methods	Search strategy	Section 2.2 + Supplementary Table S1
Methods	Selection process	Section 2.3
Methods	Data collection process	Section 2.4
Methods	Risk of bias assessment	Section 2.5 + Supplementary Table S3
Methods	Synthesis methods	Section 2.6 + Supplementary Table S4
Results	Study selection	Figure 1
Results	Study characteristics	Table 1
Results	Risk of bias in studies	Supplementary Table S3
Discussion	Limitations	Discussion section
Funding	Support and conflicts	Declarations section

Footnote. The PRISMA 2020 checklist was completed in accordance with the PRISMA 2020 reporting guideline for systematic reviews.
